# Assessment of The Factors Related to The Spontaneous Passage of Common Bile Duct Stones

**DOI:** 10.3390/jcm13092672

**Published:** 2024-05-02

**Authors:** Bayram İnan, Ahmet Akbay, İbrahim Ethem Güven, Osman Ersoy

**Affiliations:** 1 Department of Gastroenterology, Ankara City Hospital, Bilkent, Ankara 06800, Turkey; bayraminan84@gmail.com (B.İ.); drakbay@hotmail.com (A.A.); oersoy@yahoo.com.tr (O.E.); 2 Departmant of Gastroenterology, Ankara Yildirim Beyazit University Yenimahalle Training and Research Hospital, Ankara 06370, Turkey; 3 Departmant of Gastroenterology, School of Medicine, Ankara Yildirim Beyazit University, Ankara 06800, Turkey

**Keywords:** common bile duct stones, endoscopic retrograde cholangiopancreatography, magnetic resonance cholangiopancreatography, spontaneous passage of bile duct stones

## Abstract

**Background:** Common bile duct (CBD) stones may pass spontaneously without any intervention. Assessment of the predictors of spontaneous passage can contribute to avoiding unnecessary endoscopic retrograde cholangiopancreatography (ERCP) implementation. This study aimed to investigate the factors related to spontaneous passage of CBD stones. **Methods:** From January 2021 to August 2023, patients with naïve papilla who had undergone biliary ERCP and with CBD stones detected by MRCP before the procedure were analyzed retrospectively. Subjects were divided into two groups on the basis of the presence of stones during the ERCP procedure: the spontaneous passage group and the non-passage group. Groups were compared in terms of demographic, laboratory, and radiological data. **Results:** A total of 236 patients, including 26 in the spontaneous passage group and 210 in the non-passage group, were involved. Multivariate logistic regression analyses revealed that only stone size was significantly associated with spontaneous passage. From ROC curve analysis, stone size with a cut-off value of 4.3 mm predicted spontaneous passage with 58% sensitivity and 85% specificity. **Conclusions:** Stones with a size of less than 4.3 mm are more likely to pass spontaneously without endoscopic intervention. Paying attention to the stone diameter before ERCP procedures can contribute to avoiding unnecessary ERCP implementation.

## 1. Introduction

Endoscopic retrograde cholangiopancreatography (ERCP) is the gold standard technique in the management of pancreaticobiliary diseases [1,2,3]. In recent years, the number of centers where ERCP is performed and ERCP training is provided has been increasing [4]. While ERCP is thought to be a safe procedure, it can be associated with serious clinical complications, including pancreatitis, perforation, and bleeding [5,6,7]. Considering that ERCP may be associated with serious complications and due to its increasing accessibility, it becomes crucial to avoid unnecessary implementation of this procedure [8].

In clinical practice, common bile duct (CBD) stones account for the majority of indications for ERCP procedure [9]. In the diagnosis of CBD stones, magnetic resonance cholangiopancreatography (MRCP) has been widely used [10]. However, although CBD stones are detected in MRCP, stones may not be encountered during the ERCP procedure, which can be explained by the spontaneous passage of the stones [3,11]. Since CBD stones may pass spontaneously without any intervention, evaluating the predictors of this natural course can contribute to avoiding unnecessary ERCP implementation and related complications [12]. In the literature, the number of studies evaluating the factors related to spontaneous passage is insufficient, and each of these studies has a different research method, revealing conflicting results. In addition, some of these studies were performed with imaging modalities such as computed tomography and USG, which have lower sensitivity in the assessment of common bile duct stones compared to MRCP [13,14,15,16]. Therefore, literature data on this concept are lacking and further studies are warranted. 

In the presented study, we aimed to assess predictors of the spontaneous passage of CBD stones.

## 2. Materials and Methods

The presented study was carried out in a single tertiary medical center and was designed as a retrospective cohort study. Approval of this study was obtained from the local ethics committee (Approval no: 2023, E1-23-4036). Informed consent was obtained before each ERCP procedure.

### 2.1. Study Population

All consecutive patients with naïve papilla who had undergone biliary ERCP between January 2021 and August 2023 were retrospectively analyzed. Patients who were evaluated with MRCP before the ERCP procedure and with CBD stones detected as a result of the radiological examination were recruited for the study. Patients with a history of previous ERCP, undergoing ERCP with an indication other than CBD stones, altered GI anatomy, age younger than 18 years old, pancreaticobiliary anomaly such as ectopic biliary opening, and patients transferred from other hospitals due to cannulation failure or the absence of an ERCP center were excluded from the study due to the possibility of delay in diagnostic evaluation. In addition, patients with poor MRCP image quality which would affect the radiologic examination were also excluded from the study. Lastly, patients with unsuccessful cannulation were not included in the study, since ERCP success was required to determine the absence of the stone.

All ERCP procedures were carried out by an experienced ERCP team. All patients were hospitalized before the ERCP procedure. Patients without complications were discharged after 24 h observation. Patients who developed post-ERCP complications were hospitalized for an appropriate period for the management of complications.

### 2.2. Data Collection

Demographic and laboratory data of the patients, ERCP outcomes, and post-ERCP complications were recorded from the printed and electronic files of the patients retrospectively. Patients’ laboratory data, including liver function test, amylase, lipase, hemogram and C-reactive protein, were recorded from the initial laboratory findings at admission. MRCP findings including the diameter of the stone, the number of the stone, and diameter of the CBD was obtained from the radiological reports. Subjects were divided into two groups regarding the presence of the stone in the ERCP procedure: the spontaneous passage group and the non-passage group. The spontaneous passage group was defined as the absence of the stone in CBD in the ERCP session. The absence of the stone was determined by demonstrating the absence of filling defect on cholangiography and showing that the CBD was clean by using a stone extraction balloon during ERCP. Cholangiography evaluation was performed by imaging under fluoroscopy obtained by the injection of an appropriate contrast agent following successful cannulation.

### 2.3. Statistical Analysis

The normality distribution of numerical variables was analyzed by using the Kolmogorov–Smirnov test. Normally distributed numerical variables were expressed as mean ± standard deviation (SD) and were compared by using the Student’s *t*-test. Non-normally distributed numerical variables were expressed as median (interquartile range) and were compared by using the Mann–Whitney U test. Categorical variables were given as frequency (percentages) and the Chi-square test or Fisher’s exact test was used for comparisons. Univariate binary logistic regression analyses were performed for variables that may have predicted the spontaneous passage of common bile duct (CBD) stones. Afterwards, multivariate binary logistic regression analyses were performed for variables that had a *p*-value ≤ 0.1 in univariate analyses. The results were expressed as Odds ratio (OR), 95% confidence interval (CI), and *p*-value. Receiver operating characteristic (ROC) curve analyses were performed for numerical variables that statistically significantly differed in multiple variate binary logistic regression analyses. The results were given as area under curve (AUC), 95% CI, cut-off value, sensitivity, specificity, and *p*-value. The cut-off value was determined as the maximum value of the Youden index (sensitivity + specificity- 1). A *p*-value < 0.05 was considered statistically significant. IBM SPSS Statistics for Windows, version 25.0 (IBM Corp., Armonk, NY, USA) was used for analyses.

## 3. Results

A total of 236 patients were recruited for the study; 26 patients were in the spontaneous passage group and 210 patients were in the non-passage group. The baseline characteristics, laboratory, and MRCP data of the study’s cohort are detailed in Table 1. The mean age of the whole study group was 60.3 ± 17.52 years, and 109 (46.2%) of the patients were male. The mean age of the non-passage group was statistically higher than the spontaneous passage group (61.24 ± 17.31 vs. 52.69 ± 17.72, *p* = 0.019). The Charlson comorbidity index (CCI) score was higher in the non-passage group compared to the spontaneous passage group (4 (3–7) vs. 7 (5–8), *p* < 0.001). Moreover, the number and the diameter of the CBD stones were statistically lower in the spontaneous passage group (*p* = 0.047 and *p* < 0.001, respectively). Other parameters revealed no statistical difference between the groups in terms of demographic and laboratory findings.

Table 2 represents the ERCP findings and outcomes of the study groups. The median length of hospital stay was 7(6–11) days and the time from hospital arrival to ERCP was 4.5(3–6) days. Complications related to the ERCP procedure occurred in 27 patients (11.4%), and the most common ERCP-related complication was pancreatitis (*n* = 24, 10.2%). While three patients (1.3%) required intensive care follow-up, mortality occurred only in one patient (0.4%). No significant difference was detected regarding ERCP findings and outcomes between the non-passage group and spontaneous passage group (*p* > 0.05 for all parameters).

Univariate and multivariate analyses of the predictors of spontaneous passage of CBD stones are expressed in Table 3. On univariate analysis, three parameters were demonstrated to be statistically significant predictors (age, CCI, CBD stone diameter). However, only one parameter was found to be significant in predicting the spontaneous passage of CBD stones on multivariate analysis: CBD stone diameter (OR: 0.787, 95% CI: 0.642–0.963, *p* = 0.020).

**Figure 1:**
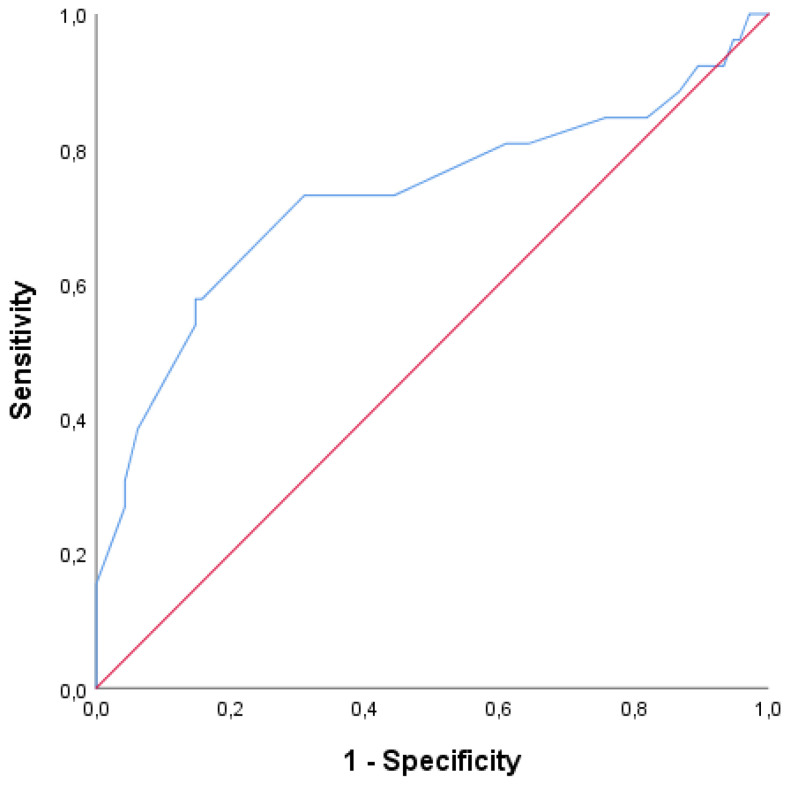
Receiver operating characteristic (ROC) curves of common bile duct (CBD) stone diameter in magnetic resonance cholangiopancreatography (MRCP) for predicting spontaneous passage of CBD stones. Red Line: Reference line, Blue Line: CBD stone diameter.

The ROC curve analysis results showing the ability of CBD stone diameter in MRCP to predict spontaneous passage are given in Table 4 and Figure 1. The diameter of the CBD stones in MRCP (with a cut-off value of 4.3 mm) predicted spontaneous passage with 58% sensitivity and 85% specificity (AUC:0.727, 95% CI: 0.600–0.854, *p* < 0.001).

## 4. Discussion

In this study, we demonstrated that the mean age, the CCI scores, the number, and the diameter of the CBD stones were higher in the non-passage group. Notably, only the CBD stone diameter was found to be significant in predicting the spontaneous passage of CBD stones in multivariate analyses, and ROC curve analysis revealed that the diameter of the CBD stones with a cut-off value of 4.3 mm predicted spontaneous passage with 58% sensitivity and 85% specificity.

ERCP plays an important role in both the evaluation and management of pancreaticobiliary diseases [3,9]. Despite the increasing awareness of the application of ERCP and technological developments in the instruments used, it can be accompanied by severe clinical complications [5,12]. The most common ERCP-related complication is post-ERCP pancreatitis with a rate of 1–10%, and when risk factors are present the rate can increase even further [17]. In this context, the first step in preventing complications can be achieved by avoiding unnecessary ERCP and accurately evaluating the indication [18]. The most common indication for ERCP procedures is CBD stones and associated cholangitis [19]. Previous studies demonstrated that CBD stones detected by radiologic evaluation may spontaneously pass through the papilla without applying ERCP [13,14,15,16]. In terms of avoiding unnecessary ERCP implementations, assessment of the factors related to the spontaneous passage of CBD stones is crucial [20].

In this study, spontaneous passage of stones was observed in 11% of the patients. In previous studies, this rate varied between 20% and 70% [16,21,22]. However, most of the previous studies used transabdominal ultrasound as a diagnostic tool before procedures, which has a low diagnostic accuracy in the assessment of bile duct stones. The low rate of spontaneous passage in the presented study can be explained by the fact that we used MRCP as a radiological evaluation before procedures and demonstrated the absence of the stone during ERCP implementation; thus, a more sensitive assessment was applied. 

Multiple factors which can be associated with the spontaneous passage of CBD stones were evaluated in previous studies. A previous study demonstrated that advanced age was associated with the non-passage of stones [15]. The mean age was also higher in the non-passage group in our study; however, age was not a significant predictor in multivariate analyses. Radiological parameters including location, number, the diameter of the stones, and the diameter of the CBD were also evaluated in previous studies. However, data regarding radiological predictive factors revealed conflicting results, yet the majority of the previous studies demonstrated that a CBD stone diameter less than 5 mm was associated with spontaneous passage [14,15,16]. Sanguanlosit et al. demonstrated that a stone size of less than 4.8 mm was more likely to pass spontaneously with 81% sensitivity and 78% specificity [16]. Khoury et al. reported that a stone size of less than 3.5 mm was a predictor of spontaneous passage with 71% sensitivity and 69% specificity [15]. In our study, we demonstrated that a stone size less than 4.3 mm was significant in predicting the spontaneous passage of CBD with 58% sensitivity and 85% specificity. In terms of the number of CBD stones, Sanguanlosit et al. reported that a single CBD stone had a higher tendency to spontaneously pass through the papilla [16]. We also found that the single stone rate was higher in the spontaneous passage group; however, on multivariate analysis, the number of the CBD stones was not found to be a predictor of spontaneous passage. On the basis of the findings of our study, paying attention to the stone diameter and performing close follow-up for patients with stones of less than 4.3 mm diameter may contribute to avoiding unnecessary ERCP processes, which will reduce ERCP-related complications and healthcare expenditure. Clinicians may consider repeat radiological examination before ERCP in patients with small CBD stones if there is a clinical doubt about whether the stone has passed or not. 

Laboratory data, especially liver function tests, were also the focus of earlier studies. Previous studies did not reveal any significant relationship between the biochemical findings at the time of the admission and the spontaneous passage of stones [14,16,23,24]. Consistent with the previous studies, liver function tests did not demonstrate any difference between the groups in our study. However, as in the previous studies, we did not evaluate the dynamic changes in biochemical data. Only one earlier study had assessed the dynamic changes in liver function tests, and demonstrated that improvements in gamma-glutamyl transferase levels were predictors of spontaneous passage [15].

The major limitation of this study was Its retrospective design. In addition, we only evaluated the laboratory parameters at the time of admission, which prevented further evaluation of changes in liver function tests as a predictive factor. Moreover, only patients with successful cannulation were included in the study, and therefore the number of participants may have been influenced by the rate of technical success. Lastly, it was a single-center study, and the study groups were not similar in terms of the number of the participants included; however, a relatively large cohort of patients were recruited in the study.

## 5. Conclusions

In conclusion, we have demonstrated that CBD stone diameter was significant in predicting the spontaneous passage of CBD stones. Since CBD stones may have a natural course in terms of spontaneous passage through the papilla, it is crucial for endoscopists to pay attention to the stone diameter before ERCP procedures in order to avoid unnecessary implementation and ERCP-related complications. Further prospective randomized studies with larger sample sizes are needed to establish the predictors of spontaneous passage of CBD stones.

## Figures and Tables

**Table 1:** Demographics, laboratory data at admission, MRCP findings of the whole study group and subgroups, and intergroup comparisons ^x^.

	Whole Study	Spontaneous Passage	Non-Passage	*p*
	Group	Group	Group	
	(*n* = 236)	(*n* = 26)	(*n* = 210)	
Age, years	60.3 ± 17.52	52.69 ± 17.72	61.24 ± 17.31	**0.019**
Gender, male, n (%)	109 (46.2)	13 (50)	96 (45.7)	0.679
CCI	2 (1–4)	1 (0–3)	2 (1–4)	**0.014**
History of cholecystectomy, n (%)	47 (19.9)	6 (23.1)	41 (19.5)	0.669
Hb level (g/dL)	13.55 (12.53–14.48)	13.45 (12.68–14.35)	13.6 (12.5–14.5)	0.872
WBC count (10^9^/L)	8.6 (6.61–10.88)	9.39 (7.29–11.68)	8.56 (6.54–10.63)	0.141
Platelet count (10^9^/L)	258 (216–308.5)	274 (215.5–320.75)	257 (215–305.5)	0.532
Total bilirubin (mg/dL)	2.45 (1.1–4.3)	2.6 (0.88–4.08)	2.4 (1.1–4.4)	0.435
Direct bilirubin (mg/dL)	1.5 (0.5–2.9)	1.4 (0.4–2.25)	1.5 (0.5–3)	0.379
Amylase (U/L)	65.5 (45–127)	98.5 (40.25–728.5)	64 (45–119.25)	0.230
Lipase (U/L)	42 (30–94.25)	54.5 (31.5–360.5)	41 (30–82)	0.217
AST (U/L)	149 (56.25–297.75)	125.5 (54.5–311.5)	150 (56.5–297.25)	0.837
ALT (U/L)	197.5 (66.25–346)	146.5 (86–312.75)	203.5 (60.5–346.5)	0.708
GGT (U/L)	363 (175–649)	270.5 (169.75–647)	371.5 (173.5–650.5)	0.322
ALP (U/L)	214 (128–320)	176 (119.25–258)	216 (130.5–323)	0.153
CRP (mg/L)	14.5 (5.27–66.95)	11.3 (5.83–75.8)	15.2 (5.12–66.38)	0.966
Distal CBD stones in MRCP, n (%)	147 (62.3)	17 (65.4)	130 (61.9)	0.730
Number of CBD stones in MRCP, n (%)				**0.047**
Single	111 (47)	17 (65.4)	94 (44.8)	
Multiple	125 (53)	9 (34.6)	116 (55.2)	
CBD diameter in MRCP (mm)	11 (9–13)	10 (7–12.25)	11 (9–13)	0.237
CBD stone diameter in MRCP (mm)	7 (5–8)	4 (3–7)	7 (5–8)	**<0.001**
CBD dilatation in MRCP, n (%)	205 (86.9)	23 (88.5)	182 (86.7)	1

^x^ Results are expressed as mean ± standard deviation, median (interquartile range), or frequency (%). Significant *p*-values are in bold. MRCP: Magnetic resonance cholangiopancreatography, CCI: Charlson comorbidity index, Hb: Hemoglobin, WBC: White blood cell, AST: Aspartate aminotransferase, ALT: Alanine aminotransferase, GGT: Gamma-glutamyl transferase, ALP: Alkaline phosphatase, CRP: C-reactive protein, CBD: Common bile duct.

**Table 2:** ERCP findings and outcomes of the whole study group and subgroups, and intergroup comparisons ^x^.

	Whole Study Group (*n* = 236)	Spontaneous Passage Group (*n* = 26)	Non-Passage Group (*n* = 210)	*p*
Length of hospital stay, days	7 (6–11)	7 (5–11.5)	7 (6–11)	0.834
Time from hospital arrival to ERCP, days	4.5 (3–6)	5 (3–6)	4 (3–6)	0.949
Time from ERCP to discharge, days	3 (1–5)	3 (1–5)	3 (1–5)	0.949
ERCP successes, *n* (%)	236 (100)	26 (100)	210 (100)	-
ERCP complications, *n* (%)	27 (11.4)	2 (7.7)	25 (11.9)	0.748
Pancreatitis	24 (10.2)	2 (7.7)	22 (10.5)	1
Bleeding	3 (1.3)	-	3 (1.4)	1
Perforation	2 (0.8)	-	2 (1)	1
Need for ICU follow-up, *n* (%)	3 (1.3)	-	3 (1.4)	1
Mortality, *n* (%)	1 (0.4)	-	1 (0.5)	1

^x^ Results are expressed as median (interquartile range) or frequency (%). ERCP: Endoscopic retrograde cholangiopancreatography, CBD: Common bile duct, ICU: Intensive care unit.

**Table 3:** Univariate and multivariate logistic regression analyses of predictors for spontaneous passage of CBD stone.

	Univariate Analysis				Multivariate Analysis			
		95% CI				95% CI		
	OR	Lower	Upper	*p*	OR	Lower	Upper	*p*
Age	0.973	0.950	0.996	**0.021**	0.993	0.946	1.042	0.779
Gender					-	-	-	-
Female	1	-	-	-				
Male	1.187	0.525	2.684	0.680				
CCI	0.747	0.578	0.965	**0.026**	0.888	0.537	1.467	0.643
History of cholecystectomy	1.237	0.467	3.275	0.669	-	-	-	-
Hb level	0.977	0.777	1.227	0.840	-	-	-	-
WBC count	1.066	0.971	1.170	0.177	-	-	-	-
Platelet count	1.002	0.998	1.006	0.345	-	-	-	-
Total bilirubin	0.905	0.755	1.086	0.284	-	-	-	-
Direct bilirubin	0.843	0.649	1.095	0.201	-	-	-	-
Amylase	1	1	1.001	0.274	-	-	-	-
Lipase	1	1	1	0.639	-	-	-	-
AST	0.999	0.997	1.001	0.568	-	-	-	-
ALT	0.999	0.997	1.001	0.474	-	-	-	-
GGT	0.999	0.998	1.001	0.291	-	-	-	-
ALP	0.998	0.995	1.001	0.188	-	-	-	-
CRP	1	0.993	1.008	0.951	-	-	-	-
Distal CBD stones in MRCP	1.162	0.495	2.732	0.730	-	-	-	-
Number of CBD stones in MRCP					0.486	0.201	1.175	0.109
Single								
Multiple	1	-	-	-				
	0.429	0.183	1.006	0.052				
CBD diameter in MRCP	0.916	0.802	1.048	0.202	-	-	-	-
CBD stone diameter in MRCP	0.742	0.609	0.904	**0.003**	0.787	0.642	0.963	**0.020**
CBD dilatation in MRCP	1.179	0.332	4.188	0.798	-	-	-	-
Time from hospital arrival to ERCP	0.983	0.867	1.115	0.792	-	-	-	-

Significant *p*-values are in bold. CBD: Common bile duct, MRCP: Magnetic resonance cholangiopancreatography, OR: Odds ratio, CI: Confidence interval, CCI: Charlson comorbidity index, Hb: Hemoglobin, WBC: White blood cell, AST: Aspartate aminotransferase, ALT: Alanine aminotransferase, GGT: Gamma-glutamyl transferase, ALP: Alkaline phosphatase, CRP: C-reactive protein, ERCP: Endoscopic retrograde cholangiopancreatography.

**Table 4:** The ability of CBD stone diameter in MRCP to predict spontaneous passage of CBD stones.

		95% CI			Cut-Off Value		
	AUC	Lower	Upper	*p*		Sensitivity	Specificity
CBD stone diameter in MRCP	0.727	0.600	0.854	**<0.001**	4.3	0.577	0.852

Significant *p*-values are in bold. CBD: Common bile duct, MRCP: Magnetic resonance cholangiopancreatography, AUC: Area under curve, CI: Confidence interval.

## Data Availability

The datasets used and/or analyzed during the current study are available from the corresponding author upon reasonable request.
